# Physico-chemical and sensory properties of cookies made from blends of germinated pigeon pea, fermented sorghum, and cocoyam flours

**DOI:** 10.1002/fsn3.2

**Published:** 2013-01-08

**Authors:** Laura Okpala, Eric Okoli, Emelem Udensi

**Affiliations:** 1 Department of Food Science and Technology, Ebonyi State UniversityAbakaliki, Nigeria; 2 Department of Food Science and Technology, Abia State UniversityUturu, Abia State, Nigeria

**Keywords:** Cocoyam, cookies, pigeon pea, sorghum

## Abstract

Cookies were produced from germinated pigeon pea, fermented sorghum, and cocoyam flour (CF) blends to determine their potentials in cookie manufacture. Ten flour formulations were produced and they were evaluated for their proximate and functional properties. Protein content ranged from 4.85% to 19.89% with 100% CF (100CF) having the least value, while 100% germinated pigeon pea flour (100GPF) had the highest value. Increase in levels of GPF to the flour blends resulted in increase in protein content of the blends. Cookies made with 100% fermented sorghum flour (100FSF) had the highest ash content of 2.73%, while cookies made with 100GPF had the least ash content. Energy values of the cookies ranged between 369.37 and 376.56 kcal/100 g, with cookie formulation 50%CF:50%FSF having the least value and cookies made with 16.7%CF:16.7%FSF:66.6GPF having the highest value. The control (cookies made with wheat) had the highest spread ratio of 24.13, while cookies made with 100FSF had the least spread ratio of 14.97. Cookies made with 100CF were the least fragile. Sensory ratings revealed that cookies containing up to 50% CF and above, compared favorably with those made with wheat flour.

## Practical Applications

The importance of this work arises from the possibility of utilizing flours from locally grown crops other than wheat to produce cookies of acceptable nutritional, sensory, and physical quality. The use of germinated pigeon pea, fermented sorghum, and cocoyam flour (CF) blends in cookie manufacture can greatly enhance the protein content, without compromising consumer acceptance.

## Introduction

Cookies are snacks that are popular and widely consumed all over the world by people of all ages. They are traditionally made from soft wheat, a cereal, which is cultivated in many parts of the world, but imported by countries with unfavorable climatic conditions. Such importing countries spend a lot of foreign exchange on importation of wheat. There is a compelling need to develop an adequate substitute for wheat, as the demand and price of this product could further be increased by natural disasters such as hurricanes, which destroy wheat crops and also the fact that many farmers are beginning to switch from growing wheat to growing “more lucrative” crops (like corn and soy beans), which could be used in the production of biofuels. In the quest for a wheat substitute, flour with better nutritional quality than wheat would be highly desirable, especially in developing countries where malnutrition is prevalent.

Composite flour can be defined as a mixture of several flours obtained from roots and tubers, cereal, legumes, etc., with or without the addition of wheat flour (Adeyemi and Ogazi 1985). Usually, the aim of producing composite flour is to get a product that is better than the individual components. Better may mean improved properties or performances, or in some cases, improved economies. The nutritional value of cereal flours that are poor in lysine but rich in the sulfur containing amino acids is improved by the addition of legume flours, and the nutritional value of root and tuber flours, which are poor in protein, is sufficiently improved by the addition of cereal flours (FAO 1990). Cereals and legumes are good sources of protein, which complement each other with respect to their amino acid profile (Ihekoronye and Ngoddy 1985). These grains are sometimes traditionally processed using methods such as fermentation and germination, which have been reported to improve their nutritional quality. Hallén et al. (2004) reported that germination induces an increase in free limiting amino acids with modified functional properties of seed components. Chavan and Kadam (1989) have also reported that fermentation of grains improves amino acid composition and vitamin content, increases protein and starch availabilities and lowers levels of anti-nutrients. It is therefore expected that composite flour produced from germinated cereals and fermented legumes will have the advantage of improving overall nutrition.

Pigeon pea and sorghum are good protein sources, but they are underutilized. Pigeon pea (*Cajanus cajan*), an important food legume, is mainly a subsistence crop in the tropics and sub-tropics of India, Africa, South-East Asia, and Central America (ICRISAT 2008). The crop is able to survive and reproduce in environments characterized by severe moisture stress and poor soil fertility. It is cultivated as a sole crop or intermixed with such cereals as sorghum, millet, or maize. It also contains about 25.83% of protein (Tiwari et al. 2008).

Sorghum (*Sorghum bicolor*), a cereal, is a member of the grass family. It is the fifth most important cereal in the world after wheat, rice, corn, and barley. More than 35% of sorghum is grown for human consumption, while the rest is primarily used for feed and industrial alcohol production (Awadelkareem et al. 2009). Available data from FAO (1988) report that there is a decrease in consumption in parts of Africa, and this has been attributed to shift in consumer habits brought about by rapid rate of urbanization, time and energy required to prepare food based on sorghum, as well as poor marketing facilities and processing techniques.

Cocoyam (*Xanthosoma sagittifolium*) is an edible root crop grown in the tropics of which Nigeria is a major producer. It belongs to the family Aracea (Ihekoronye and Ngoddy 1985). The flour from cocoyam has been used in baking of products as it has been reported that cocoyam has fine granule-starch, which improves binding and reduces breakage of snack products (Huang 2005).

As there is a growing interest in the production of flours from locally available grains that can be used as substitutes for wheat in baked goods, this study was undertaken to produce cookies of acceptable quality from germinated pigeon pea, fermented sorghum, and CF blends.

## Materials and Methods

The white variety of pigeon pea (*C. cajan*), the white variety of sorghum (*S. bicolor*), and the tannia variety of cocoyam (*X. sagittifolium)* were purchased from a retail outlet in Abakaliki, Ebonyi State, Nigeria. Wheat flour and all other baking ingredients such as eggs, baking powder, fat, milk, and flavorings were also obtained from the same source.

### Sample preparation

#### Cocoyam flour

CF was produced using the method described by Udensi et al. (2008) with slight modifications. The corms were washed, peeled, sliced, and blanched at 80°C for 4 min. They were dried and milled to pass through 100-μm mesh sieve.

#### Germinated pigeon pea flour (GPF)

Pigeon peas were germinated using the modified method of Hallén et al. (2004). Cleaned grains were soaked in 0.1% sodium hypochlorite solution for 30 min to prevent mold growth. The grains were thereafter thoroughly washed and soaked in water (10 h). The hydrated seeds were spread on jute bags and allowed to germinate for 4 days after which they were dried at 50°C. Thereafter, formed roots and testa were rubbed off before milling and sieving through 100-μm mesh sieve.

#### Fermented sorghum flour (FSF)

The sorghum flour was subjected to natural lactic acid fermentation using the method of Hallén et al. (2004). Washed and dried grains were ground into fine flour. The flour was mixed with water (1:5 wt/wt) to form slurry followed by the addition of 5% sugar by weight of flour. The slurry was left to ferment in trays at room temperature for 4 days when the pH of the slurry reached 5.5. The fermented slurry was dried at 50°C and then ground through a 100-μm mesh screen. All of the flour samples were kept in airtight containers until needed for analysis.

### Cookie formulation and preparation

The ratio of ingredients used in the preparation of the cookies is presented in Table 1, while the cookie formulations are shown in Table 2. The ingredients used were: flour, 100.0 g; hydrogenated vegetable fat, 40.0 g; sugar (granulated cane), 25.0 g; egg (whole, fresh), 31.0 g; milk (full-fat filled, powdered), 7.8 g; nutmeg, 0.3 g; vanilla (liquid), 5.0 mL; salt, 1.0 g; and baking powder, 1.0 g. Fat and sugar were creamed using an electric mixer at medium speed for 5 min. Eggs and milk were added while mixing and then mixed for a total of about 30 min. Vanilla, nutmeg, flour, baking powder, and salt were mixed thoroughly and added to the cream mixture where they were all mixed together to form a dough. The dough was kneaded to a uniform thickness of 0.25 cm and cut into circular shapes of 5 cm diameter. Baking was carried out at 185°C for 20 ± 5 min. Cookie samples were cooled and stored in airtight containers until needed. Cookies were made from wheat to serve as a control.

**Table 1:** Ratio of ingredients for cookies

Ingredients	Amount (% w/w)
Flour^1^	46.7
Hydrogenated vegetable fat	18.7
Sugar	11.7
Milk	7.0
Baking powder	0.5
Vanilla flavor	0.2
Nutmeg	0.1
Salt	0.5
Egg	14.6

1 Wheat, pigeon pea, cocoyam, sorghum, or composite flour.

**Table 2:** Proximate composition of germinated pigeon pea, fermented sorghum, and blanched cocoyam flour blends

Blends (CF:FSF:GPF)	Moisture (%)	Fat (%)	Protein (%)	Ash (%)	Fiber (%)	Carbohydrates (%)
100:0:0	8.05^d^	1.76^c^	4.85^i^	2.10^b^	1.46^e^	81.78^a^
0:100:0	7.45^f^	1.68^d^	8.08^g^	1.96^e^	1.65^a^	79.18^b^
0:0:100	7.25^g^	1.55^e^	19.89^a^	1.92^e^	1.56^c^	67.82^h^
50:50:0	8.11^c^	1.83^b^	7.45^h^	1.87^f^	1.47^de^	79.26^b^
0:50:50	7.16^h^	1.64^d^	16.23^c^	2.09^bc^	1.59^bc^	72.08^g^
50:0:50	6.95^i^	1.56^e^	14.83^d^	2.11^b^	1.65^a^	72.90^f^
33.3:33.3:33.3	8.25^b^	1.47^f^	9.10^f^	2.06^cd^	1.65^a^	77.47^d^
16.7:16.7:66.6	7.77^e^	1.86^b^	16.23^b^	2.17^a^	1.62^ab^	70.36^h^
16.7:66.6:16.7	8.51^a^	1.66^d^	11.74^e^	2.02^d^	1.56^c^	74.50^e^
66.6:16.7:16.7	6.68^j^	1.94^a^	9.12^f^	2.05^cd^	1.51^d^	78.69^c^

CF, cocoyam flour; FSF, fermented sorghum flour; GPF, germinated pigeon pea flour.

Means within a column with the same superscript are not significantly different (*P* > 0.05).

Values in Tables 2–4 are means of triplicate determinations.

### Proximate composition of flour blends and cookies

Protein, fat, ash, fiber, and moisture contents of flour blends and cookies were determined according to the methods described by AOAC (1990). Carbohydrates were determined by difference. Energy was calculated by the Atwater method (Osborne and Voogt 1978). Analyses were performed in triplicate.

### Functional properties of flour blends

The oil and water absorption capacities were determined according to the methods described by Beauchat (1977). Bulk density and emulsion capacity were determined as described by Onwuka (2005). Analyses were performed in triplicates.

### Physical analysis of cookies

Cookies diameter and thickness were determined using vernier callipers, while cookies weight was determined using an electronic weighing balance. Spread ratio was expressed as diameter/thickness (McWatters et al. 2003). Fragility was determined using the method described by Okaka and Isieh (1990). A representative sample of cookies from each formulation (of same average weight) was placed centrally between two parallel wooden bars (3 cm apart) with dimensions 21 × 13 × 0.70 cm. Another wooden bar of dimensions 21 × 13 × 2.0 cm (0.3 kg) was placed on the cookie. Weights were then placed on the bar incrementally until the cookie fractured. The least weight that caused the breaking of the cookie was regarded as the fragility of the cookie. Ten representative samples were analyzed from each formulation.

### Sensory evaluation

Twenty-four hours after preparation of the cookies, sensory evaluation was carried out. A total of 20 semi-trained panelists were recruited from staff and students of the Ebonyi State University, Abakaliki. Each panelist evaluated all the samples prepared for each treatment in one session. Criteria for selection of panelists were that panelists were regular consumers of cookies and were not allergic to any food. Panelists were instructed to evaluate appearance, taste, texture, crispness, and general acceptability of the cookies. A nine-point Hedonic scale was used with 1 = dislike extremely, 5 = neither like nor dislike, and 9 = like extremely (Ihekoronye and Ngoddy 1985). Samples were identified with three-digit code numbers and presented in a random sequence to panelists. The panelists were instructed to rinse their mouths with water after every sample and not to make comments during evaluation to prevent influencing other panelists. They were also asked to comment freely on samples on the questionnaires given to them.

### Statistical analysis

The data collected were subjected to analysis of variance (ANOVA). Means were separated using Duncan's multiple range test using the Statistical Package for the Social Sciences (SPSS) version 16.0 (SPSS Inc., Chicago, IL).

## Results and Discussion

### Proximate composition of flour blends and cookies

The proximate composition of the flour samples is shown in Table 2. The 100% GPF (100GPF) had the highest protein content of 19.89%. This was followed by the flour blend of 16.7% cocoyam, 16.7% fermented sorghum, and 66.6% germinated pigeon pea (16.7CF:16.7FSF:66.6GPF). The high-protein content of these flour samples is because pigeon pea has been reported to be high in protein (Tiwari et al. 2008). Hundred percent CF (100CF) had the least protein and fiber contents, but had the highest carbohydrate content. The observed increase in carbohydrate content of flour blends containing CF could be due to the high content of carbohydrate in cocoyam. According to Enwere (1998), of all the solid nutrients in roots and tubers, carbohydrates predominate.

Table 3 shows the proximate composition of the cookies produced. The moisture contents of the cookies were generally low; values were less than 10%, and as such, moisture in the cookies is unlikely to cause any adverse effect on the quality attributes of the product. Cookies made with 100GPF had the highest protein content of 21.43%. Okpala and Okoli (2011) reported that cookies made with 100% pigeon pea flour (ungerminated) had a protein content of 12.97%. This suggests that germination of the pigeon pea had a positive effect on the protein content of the cookies. With the exception of cookies made with 100CF and 50CF:50FSF, all the cookies had significantly higher (*P* < 0.05) protein content than the control (cookies made with wheat flour). Cookies made with 50CF:50FSF had higher carbohydrates content than cookies made with the composites. The control, however, had the highest carbohydrates content. The fat content of the cookies were generally low and this is likely to be desired by weight watchers. The fiber contents of all the cookies were within the recommended range of not more than 5 g dietary fiber per 100 g dry matter (FAO/WHO 1994).

**Table 3:** Chemical composition of cookies produced from germinated pigeon pea, fermented sorghum, and blanched cocoyam flour blends

Blends (CF:FSF:GPF)	Moisture (%)	Fat (%)	Protein (%)	Ash (%)	Fiber (%)	Carbohydrates (%)	Energy (kcal/100 g)
100:0:0	9.93^d^	6.05^de^	7.37^j^	2.65^ab^	2.28^d^	71.72^a^	370.81
0:100:0	10.07^c^	6.84^a^	9.93^h^	2.73^a^	2.27^d^	68.16^c^	373.92
0:0:100	10.15^bc^	6.15^c^	21.43^a^	2.37^e^	2.13^e^	57.77^g^	372.15
50:50:0	10.23^ab^	6.01^ef^	8.86^i^	2.56^bc^	2.38^c^	69.96^b^	369.37
0:50:50	9.83^e^	6.25^b^	17.61^c^	2.53^d^	2.46^a^	61.32^ef^	371.97
50:0:50	10.26^a^	6.09^d^	16.82^d^	2.39^e^	2.26^d^	62.18^e^	370.81
33.3:33.3:33.3	10.15^b^	6.22^b^	15.37^g^	2.64^ab^	2.46^a^	63.15^d^	370.06
16.7:16.7:66.6	9.41^f^	5.96^f^	19.61^b^	2.53^cd^	2.37^c^	61.12^f^	376.56
16.7:66.6:16.7	9.82^e^	6.15^c^	16.10^e^	2.45^de^	2.26^d^	63.22^d^	372.63
66.6:16.7:16.7	9.94^d^	6.21^b^	15.73^f^	2.45^de^	2.44^ab^	63.23^d^	371.73
100% wheat	7.46^g^	5.64^g^	9.65^i^	2.56^c^	2.41^bc^	72.28^a^	378.48

CF, cocoyam flour; FSF, fermented sorghum flour; GPF, germinated pigeon pea flour.

Means within a column with the same superscript are not significantly different (*P* > 0.05).

### Functional properties of flour blends

The functional properties of the flour blends are presented in Table 4. The bulk density of the samples ranged from 0.51 to 0.63 g/cm^3^. Flour blend 33.3CF:33.3FSF:33.3GPF had the highest value, while 16.7CF:16.7FSF:66.6GPF had the least value. The bulk density of a food material is important in relation to its packaging (Bello and Okezie 1982). Increase in bulk density is desirable, in that it offers greater packaging advantage as greater quantity may be packed within constant volume (Molina et al. 1983). The water absorption capacities of the blends were between 133.33% and 200%. The values were generally high. Water absorption capacity is the ability of a product to associate with water under limiting conditions (Singh 2001). It has been suggested that flours with such high water absorption capacity as seen in this study will be very useful in bakery products, as this could prevent staling by reducing moisture loss. The oil absorption capacities of the flour blends were generally high. Oil absorption capacity (OAC) is the ability of flour to absorb oil, which is important as oil acts a flavor retainer and improves mouth feel (Aremu et al. 2007). The high OAC suggests the lipophilic nature of the constituents of the flour (Ubbor and Akobundu 2009), and this suggests that the blends are potentially useful in structural interaction in food especially in flavor retention, improvement of palatability, and extension of shelf life of bakery or meat products, doughnuts, baked goods, pan cakes, and soup mixes where fat absorption is desired (Seena and Sridhar 2005). The emulsion capacities of the flour blends ranged from 15.67% to 46.33%. High-emulsion capacity is an indication that a flour sample could be an excellent emulsifier (Akobundu et al. 1982). Emulsifiers are incorporated into cookie formulas to improve dough handling and the product's overall quality (Hoque et al. 2009).

**Table 4:** Functional properties of germinated pigeon pea, fermented sorghum, and blanched cocoyam flour blends

Blends (CF:FSF:GPF)	Bulk density (g/cm^3^)	Water absorption capacity (%)	Oil absorption capacity (%)	Emulsion capacity (%)
100:0:0	0.58^bcd^	173.33^b^	187.00^bc^	35.52^b^
0:100:0	0.58^bcd^	133.33^c^	181.33^bc^	42.67^a^
0:0:100	0.53^ef^	200.00^a^	147.33^d^	36.00^b^
50:50:0	0.57^cde^	153.33^bc^	133.33^e^	16.67^de^
0:50:50	0.54^def^	200.00^a^	203.33^bc^	15.67^e^
50:0:50	0.57^cde^	166.67^b^	153.33^cd^	46.33^a^
33.3:33.3:33.3	0.63^a^	160.00^bc^	280.00^a^	34.67^b^
16.7:16.7:66.6	0.51^f^	200.00^a^	173.33^bcd^	33.00^b^
16.7:66.6:16.7	0.61^abc^	153.33^bc^	196.67^bcd^	45.33^a^
66.6:16.7:16.7	0.52^f^	153.33^bc^	156.67^cd^	26.00^c^

CF, cocoyam flour; FSF, fermented sorghum flour; GPF, germinated pigeon pea flour.

Means within a column with the same superscript are not significantly different (*P* > 0.05).

### Physical properties of cookies

The results of various physical properties of cookies are shown in Table 5. Significant differences (*P* < 0.05) were observed in the weight, thickness, diameter, and spread ratio of the control and some of the cookies made with the composite flours. Cookies from wheat had the highest spread ratio and it was not significantly different from three of the formulations. Cookies having higher spread ratios are considered the most desirable (Kirssel and Prentice 1979). Cookies made with 100FSF had the least spread ratio, and this suggests that the starches in 100FSF were very hydrophilic (Yahya 2004). It was observed that cookies made with 100FSF, which had the least spread ratio, also had the highest thickness. Chinma and Gernah (2007) observed a similar trend in cookies produced from cassava/soyabean/mango composite flours and attributed it to the hydrophilic nature of the flour used in producing the biscuit, which caused reduction in spread, thus leading to an increase in thickness of the cookies. Cookies made with 100CF were the least fragile.

**Table 5:** Physical properties of cookies produced from germinated pigeon pea, fermented sorghum, and blanched cocoyam flour blends

Blends (CF:FSF:GPF)	Weight (g)	Thickness (cm)	Diameter (cm)	Spread ratio	Fragility (kg)
100:0:0	7.14^d^	0.26^b^	4.81^a^	19.46^b^	3.32^a^
0:100:0	9.80^ab^	0.31^a^	4.60^bcd^	14.97^c^	0.58^d^
0:0:100	9.07^bc^	0.22^bc^	4.68^bcd^	21.28^ab^	0.50^d^
50:50:0	8.57^c^	0.25^b^	4.57^d^	18.67^bc^	1.20^c^
0:50:50	9.69^ab^	0.27^b^	4.59^cd^	17.93^bc^	1.30^c^
50:0:50	9.12^bc^	0.22^bc^	4.69^bc^	21.38^ab^	1.30^c^
33.3:33.3:33.3	9.22^bc^	0.25^b^	4.72^ab^	19.08^b^	1.00^cd^
16.7:16.7:66.6	9.79^ab^	0.25^b^	4.71^ab^	19.55^b^	2.24^b^
16.7:66.6:16.7	9.35^bc^	0.25^b^	4.58^cd^	19.96^b^	1.24^c^
66.6:16.7:16.7	9.06^bc^	0.24^b^	4.72^ab^	20.58^ab^	1.60^c^
100% wheat flour	10.39^a^	0.20^c^	4.56^d^	24.13^a^	1.50^c^

CF, cocoyam flour; FSF, fermented sorghum flour; GPF, germinated pigeon pea flour.

Means within a column with the same superscript are not significantly different (*P* > 0.05).

### Sensory evaluation

Table 6 shows sensory scores for the cookies produced. Cookies made with wheat had the highest ratings for all the sensory parameters tested. There were no significant differences (*P* > 0.05) between the ratings given to the control and cookies made with 100CF for all the sensory attributes. With the exception of general acceptability, cookies made with 50CF:50FSF and 66.6CF:16.7FSF:16.7GPF were not significantly different (*P* > 0.05) from the control. While the addition of CF improved ratings for the cookies, the addition of GPF had the opposite effect. All the cookies containing at least 50% GPF (with the exception of 16.7CF:16.7FSF:66.6GPF) received low scores (≤6) for texture, taste, crispness, and general acceptability. The results of the sensory analysis showed that texture data for cookies from 100GPF were in good agreement with the measurement derived from the fragility tests. Panelists described cookies containing high levels of GPF as having a bitter aftertaste and lacking the characteristic crispness and texture associated with cookies. This infers that even if cookies containing GPF have high protein content, many people may not be willing to eat them because of the low sensory quality. Except for those formulations containing above 50% GPF, all the cookies were at least slightly liked for the parameters studied.

**Table 6:** Sensory evaluation scores of cookies produced from germinated pigeon pea, fermented sorghum, and blanched cocoyam flour blends

Blends (CF:FSF:GPF)	Texture	Taste	Appearance	Crispness	General acceptability
100:0:0	7.6^a^	7.8^ab^	7.6^ab^	7.5^a^	7.4^ab^
0:100:0	6.3^cd^	6.5^bcd^	6.6^bcd^	6.2^cde^	7.1^b^
0:0:100	5.9^d^	5.0^e^	5.9^d^	5.3^e^	5.0^d^
50:50:0	7.1^abc^	7.1^abc^	7.0^abcd^	6.9^abcd^	7.1^b^
0:50:50	5.9^d^	6.0^cde^	6.4^cd^	5.0^e^	5.0^d^
50:0:50	5.9^d^	5.9^cde^	6.7^bcd^	5.4^e^	5.5^cd^
33.3:33.3:33.3	7.1^abc^	6.9^abcd^	6.9^abcd^	6.8^abcd^	6.8^bc^
16.7:16.7:66.6	6.6^cd^	5.6^de^	6.8^bcd^	6.0^de^	5.8^cd^
16.7:66.6:16.7	6.7^bcd^	6.7^bcd^	6.9^abcd^	6.5^bcd^	6.5^bcd^
66.6:16.7:16.7	7.5^ab^	7.5^ab^	7.6^ab^	7.1^abc^	7.2^b^
100% wheat flour	8.0^a^	8.2^a^	8.0^a^	7.7^a^	8.4^a^

CF, cocoyam flour; FSF, fermented sorghum flour; GPF, germinated pigeon pea flour.

Means within a column with the same superscript are not significantly different (*P* > 0.05).

## Conclusion

This study has shown that acceptable cookies of high-protein content can be produced from flour blends of cocoyam, germinated pigeon pea, and fermented sorghum. Several of the blends produced cookies that compared favorably with cookies from wheat flour (with respect to most of the sensory attributes). The use of these locally grown crops will go a long way in reducing dependence on wheat flour, thereby reducing foreign exchange used in importing wheat.
